# Boar Sperm Cryopreservation Improvement Using Semen Extender Modification by Dextran and Pentaisomaltose

**DOI:** 10.3390/ani12070868

**Published:** 2022-03-30

**Authors:** Ondrej Simonik, Filipa Bubenickova, Lucie Tumova, Michaela Frolikova, Vishma Pratap Sur, Jan Beran, Katerina Havlikova, Lenka Hackerova, Daniela Spevakova, Katerina Komrskova, Pavla Postlerova

**Affiliations:** 1 Laboratory of Reproductive Biology, Institute of Biotechnology of the Czech Academy of Sciences, BIOCEV, Prumyslova 595, 25250 Vestec, Czech Republic; ondrej.simonik@ibt.cas.cz (O.S.); michaela.frolikova@ibt.cas.cz (M.F.); vishmapratap.sur@ibt.cas.cz (V.P.S.); daniela.spevakova@ibt.cas.cz (D.S.); katerina.komrskova@ibt.cas.cz (K.K.); 2 Department of Veterinary Sciences, Faculty of Agrobiology, Food and Natural Resources, University of Life Sciences Prague, Kamycka 129, 16500 Prague, Czech Republic; bubenickovaf@gmail.com (F.B.); tumovalucie@af.czu.cz (L.T.); havlikovak@af.czu.cz (K.H.); hackerova@af.czu.cz (L.H.); 3 Department of Zootechnical Sciences, Faculty of Agriculture, University of South Bohemia in Ceske Budejovice, Studentska 1668, 37005 Ceske Budejovice, Czech Republic; jberan02@zf.jcu.cz; 4 Department of Zoology, Faculty of Science, Charles University, Vinicna 7, 12844 Prague, Czech Republic

**Keywords:** cryopreservation, boar sperm, dextran, pentaisomaltose, polysaccharide, reproduction, glycerol, AWN spermadhesin

## Abstract

**Simple Summary:**

Cryopreservation of sperm is an important process used in livestock breeding. It is used to facilitate artificial insemination and helps to maintain genetic diversity by utilizing storage in cryobanks. Boar sperm are extremely sensitive to the conditions present during cryopreservation. Glycerol is the most used permeable cryoprotectant, showing both protective activity and some level of sperm toxicity. To improve the insemination rates, it is necessary to develop new cryoprotective agents. The goal of this paper is to evaluate the effect of the highly biocompatible, non-toxic polysaccharides dextran and pentaisomaltose on the cryopreservation of boar sperm. Additionally, we used computational modeling for the prediction of their cryoprotective action through interaction with selected sperm surface proteins. Our results show a lower impact of cryopreservation on sperm qualitative parameters when the extender is modified by pentaisomaltose. This approach represents a promising option for the complete removal of toxic glycerol in cryopreservation.

**Abstract:**

The long-term storage of boar sperm presents an ongoing challenge, and the modification of the cryoprotective compounds in semen extenders is crucial for improving cryopreservation’s success rate. The aim of our study was to reduce the percentage of glycerol in the extender by elimination or substitution with biocompatible, non-toxic polysaccharides. For boar semen extender improvement, we tested a novel modification with the polysaccharides dextran and pentaisomaltose in combination with unique in silico predictive modeling. We targeted the analysis of in vitro qualitative sperm parameters such as motility, viability, mitochondrial activity, acrosome integrity, and DNA integrity. Non-penetrating polysaccharide-based cryoprotective agents interact with sperm surface proteins such as spermadhesins, which are recognized as fertility markers of boar sperm quality. The in silico docking study showed a moderate binding affinity of dextran and pentaisomaltose toward one specific spermadhesin known as AWN, which is located in the sperm plasma membrane. Pentaisomaltose formed a hydrophobic pocket for the AWN protein, and the higher energy of this protein–ligand complex compared with dextran was calculated. In addition, the root mean square deviation (RMSD) analysis for the molecular dynamics (MD) of both polysaccharides and AWN simulation suggests their interaction was highly stable. The in silico results were supported by in vitro experiments. In the experimental groups where glycerol was partially or entirely substituted, the use of pentaisomaltose resulted in improved sperm mitochondrial activity and DNA integrity after thawing when compared with dextran. In this paper, we demonstrate that pentaisomaltose, previously used for cryopreservation in hematopoietic stem cells, represents a promising compound for the elimination or reduction of glycerol in extenders for boar semen cryopreservation. This novel approach, using in silico computer prediction and in vitro testing, represents a promising technique to help identify new cryoprotectants for use in animal breeding or genetic resource programs.

## 1. Introduction

Gamete cryopreservation represents an important element in reproductive biotechnology. It allows for an infinitely long storage time of valuable genetic material for further use in breeding or national animal genetic conservation programs. However, the cryopreservation of boar sperm is not commonly used in practice, and only 1% of all artificial inseminations worldwide are performed using frozen-thawed doses of boar sperm [1].

The quality of boar semen and its freezability vary greatly between breeds, individual animals, and also the season of the year [2]. Damage to boar spermatozoa by cryopreservation results in a significantly lower survival rate. This phenomenon is more significant in boars than in other livestock species, where cryopreservation is a commonly used method for storing sperm. The level of damage is related to the different compositions of cryopreservation media, such as the addition of various compounds to freezing and thawing extenders like antioxidants. Boar spermatozoa are extremely sensitive to low temperatures and cold shock [3,4]. Sperm freezing is associated with the formation of extracellular ice crystals, which form during the freezing process from extracellular water. These ice crystals increase the concentration of solutes in the diluent and create an osmotic gradient on the sperm membrane, resulting in cellular dehydration [5,6,7]. Osmotic stress caused by the formation of extracellular ice crystals results in membrane damage and cell death [8]. To minimize sperm cell damage during cryopreservation, cryoprotectants are used to provide protection against cold shock. However, they can significantly affect the physiological state of the cells after thawing [9].

Cryoprotective agents can be divided into permeating and non-permeating agents according to their mechanism of action [7,10,11,12,13]. Permeating agents are generally more effective than non-permeating cryoprotectants [12,13]. Permeating agents replace water inside the cells. These cryoprotectants permeate the cell membrane more slowly than water, resulting in a transient change in osmotic pressure that can lead to cell damage. Human and bovine sperm are more resistant to these changes than boar sperm. As a result, it is important to adjust the composition of the cryoprotectant to improve sperm protection and to simultaneously minimize cell damage [13].

Glycerol is a commonly used cryoprotective permeating agent which penetrates the sperm membrane [7,10,11]. Glycerol replaces water in the cell, thus preventing the formation of crystals and interior damage to the sperm head. However, glycerol is toxic to sperm in high doses [14,15]. Glycerol toxicity is associated with osmotic stress, possible changes in membrane organization, permeability, and fluidity defects [16]. In addition, it also does not protect the mitochondria from damage during freezing and thawing [17] and adversely affects successful fertilization in many species, including poultry [15,18], sheep [17,19], pigs [17,20], and horses [21]. The adverse effect (toxicity) of glycerol is highly species-dependent, and therefore the goal is to reduce the volume of glycerol in the media or, ideally, completely replace it with another less or non-toxic cryoprotectant.

Non-permeating cryoprotectants do not pass through the plasma membrane of sperm and act extracellularly [22], and these include sugars, amino acids, various proteins, lipoproteins, and other macromolecules such as methylcellulose, polyvinyl alcohol, and polyvinylpyrrolidone [12,13]. The interaction of these cryoprotectants with the components of the plasma membrane contributes to its stabilization. However, they can cause cellular dehydration since they increase the osmolality of the cryopreservation medium [12]. The results of our previous studies [23,24] revealed the interaction of dextran sulphate with sperm surface proteins, and the strongest affinity was found to be with AWN spermadhesin. Due to their function, proteins belonging to the spermadhesin family are regarded as candidate markers of boar fertility [25,26]. The binding of polysaccharides to these proteins can represent a new way to conserve sperm function during cryopreservation. In silico modelling and molecular docking can play a crucial role in aiding the understanding of protein binding affinity and the mechanisms of binding stability. Docking and modeling also provide a method for understanding the structural basis for protein–ligand binding affinity. Additionally, knowledge of the 3D structure of a protein is very relevant for understanding the key functional roles of the protein in biological systems. During the last decade, molecular dynamics (MD) simulation has provided us with molecular insight on protein–ligand stability, compactness, hydrophobicity, and involved energy for the affinity. In reproductive biology, an in silico methodology can provide an effective way to improve resolution in terms of protein–ligand and protein–drug interactions and moreover the dynamics of protein–ligand complexes [27].

Recently, bio-inspired biocompatible materials such as ectoine and trehalose have been utilized to cryopreserve other human cell types [28]. The polysaccharide dextran belongs to the same group of compounds regarding non-toxicity and biocompatibility. Additionally, its cryoprotective effect has been demonstrated for various cell types, including stem cells [29,30,31]. Moreover, a derivate of dextran—pentaisomaltose, commercially available under the name PentaHibe^®^ (Pharmacosmos, Denmark), is a carbohydrate-based alternative for dimethyl sulfoxide (DMSO) usage in the cryopreservation of hematopoietic stem cells and T-cells [29]. In reproductive biotechnology, dextran has been tested for improving cryopreservation protocols for poultry and rabbit sperm [15,18]. For the cryopreservation of turkey sperm, dextran was used for the entire elimination of glycerol, and this discovery was the inspiration for this study.

The aim of this study was to evaluate the selected polysaccharides for their potential as alternatives to the use of glycerol or a reduction in its concentration in freezing extenders for boar sperm. The polysaccharides’ protective effects were evaluated using several in vitro functional tests, such as sperm motility and viability, as well as the integrity of the sperm acrosome, mitochondria, and DNA. For the first time in sperm cryobiology, we used an in silico experimental approach using molecular docking and MD simulation for the prediction of cryoprotectant interaction with proteins that are localized on the sperm plasma membrane.

## 2. Materials and Methods

Unless otherwise noted, all chemicals were purchased from Sigma-Aldrich^®^ (St. Louis, MO, USA).

### 2.1. In Silico Prediction of Dextran and Pentaisomaltose Interaction with Sperm Surface Protein

#### 2.1.1. Protein Structure Preparation and Molecular Docking

We assumed that the dextran ligand was interacting with AWN spermadhesin. There is no solved crystal structure for AWN spermadhesin. To obtain the 3D protein structure, we used the AWN spermadhesin fasta sequence (CAI05910.1) from the data bank using the SWISS MODELER server. AutoDock Vina 1.1.2 with PyRx 0.8 software was used to predict the AWN spermadhesin interaction with dextran and pentaisomaltose. Both the protein and ligand structures were loaded to PyRx as macromolecules and ligands, respectively, which were then converted to PDBQT files for docking. We performed blind docking with a 3D affinity grid box. The grid box was centered to cover the active site residues with dimensions x = −59.47 Å, y = 38.78 Å, and z = 23.82 Å. The size of the grid wherein all the binding residues were fitted had the following dimensions: x = 26.56 Å, y = 25.00 Å, and z = 34.61 Å. The detailed follow-up analyses for the interaction of amino acids with the ligand were performed with BIOVIA Discovery Studio Visualizer [27].

#### 2.1.2. MD Simulation

The best protein–ligand complexes were chosen for MD simulation according to the lowest binding energy with the best docked pose. Additional binding interactions were used for molecular simulation studies. MD simulation was conducted for 50 ns according to a previous study [27,32], where the simulation process was conducted using the GROMACS 2020 package, utilizing a charmm36 all-atom force field using empirical, semi-empirical, and quantum mechanical energy functions for the molecular systems. The topology and parameter files for the input ligand files were generated on the CGenff server (http://kenno.org/pro/cgenff/, accessed on 10 December 2021). The trajectory files were analyzed using GROMCAS tools—gmx rmsd, gmx gyrate, gmx sasa, gmx hbond, and gmx energy—for extracting the plot of the root mean square deviation (RMSD), root mean square fluctuations (RMSFs), radius of gyration (Rg), solvent accessible surface area (SASA), and hydrogen bond (H-bonds) [27,32].

### 2.2. Semen Collection

Ejaculates were obtained from the boar insemination center Liprapork Inc. in Skršín, Czech Republic using four Duroc breed boars that were about 2 years old. Every boar was collected four times in a period from January to May 2021. Pooling of the semen from individual boars was not performed. This company is routinely used for commercial production of short-term preserved insemination doses. Samples were collected by the gloved hand method, and semen was processed according to the standard procedures of the laboratory. Only the samples which passed a standard quality assessment were used in this study. Cryopreservation was performed according to a protocol routinely used in the laboratory for preservation of national gene resources of pig breeds in Kostelec nad Orlicí [33].

### 2.3. Sperm Cryopreservation

The ejaculate was extended at a semen dilution rate of 1:1.5 in Androhep (Minitube GmbH, Tiefenbach, Germany) and delivered to the laboratory in a digitally controlled thermo-box c at 17 °C. According to the methodology [33], the day after collection, the extended ejaculate was centrifuged at 1800× *g* for 10 min at 17 °C, and the spermatozoa were gently resuspended in the various semen extenders for cryopreservation according to the experimental design. The basic composition of the semen extender was fructose (20 g), sodium bicarbonate (0.352 g), L-cysteine (0.035 g), Lincomycin (150 μg/mL, Spectinomycin (300 μg/mL), Streptomycin (500 UI/mL), Peniciline (500 UI/mL), Equex Orvus STM Paste (3.976 g; Minitube GmbH, Tiefenbach, Germany), egg yolk (80 mL), and Milli-Q water (272 mL). The pH was adjusted to 7.3, and the osmolality of the media was in the range of 707–863 mmol/kg according to the experimental group. The following experimental groups were prepared: G3 (control with 3% glycerol), G2D1 (10) (2% glycerol, 1% dextran 10 kDa; Carl Roth GmbH + Co. KG, Karlsruhe, Germany), G2D1 (20) (2% glycerol, 1% dextran 20 kDa; Carl Roth GmbH + Co. KG), G1P5 (1% glycerol, 5% pentaisomaltose/PentaHibe^®^; Pharmacosmos, Holbæk, Denmark), and G0P10 (0% glycerol, 10% pentaisomaltose/PentaHibe^®^). The samples were subsequently loaded into 0.5-mL plastic medium straws (IMV Technologies, L’Aigle, France), equilibrated for 2 h at 5 °C, and horizontally cryopreserved in a Styrofoam box (Animal Reproduction systems, Chino, CA, USA) at 4 cm above the liquid nitrogen level for 20 min. Afterward, they were plunged directly into liquid nitrogen and stored for at least 1 week before analyses.

All samples were thawed in a circulating water bath with circulation at 37 °C for 30 s and diluted with DPBS without calcium and magnesium ions at a final concentration of 1–10 × 10^6^/mL. After washing 2 times at 300× *g* for 5 min, the samples were processed for analysis of different parameters.

### 2.4. In Vitro Analyses of Sperm Qualitative Parameters

#### 2.4.1. Sperm Motility by Computer-Assisted Sperm Analysis (CASA)

Analysis of motion parameters of post-thawed sperm was carried out using the CASA system (ISAS, Proiser, Valencia, Spain). The final concentration of spermatozoa in the samples was 20 × 10^6^ spermatozoa/mL [34]. After preincubation at 37 °C for 5 min, 2 μL of the sample was evaluated in a pre-warmed Leja counting chamber (20 μm deep) in 6 different fields per sample. The fields were strictly located at the outer edges of the chamber to avoid the Segre–Silberberg effect. On average, 200 trajectories per field were analyzed. The following kinematic parameters were derived and analyzed by cluster analysis: curvilinear velocity (VCL, μm/s), velocity of the average path (VAP, μm/s), straight line velocity (VSL, μm/s), straightness (STR, %), linearity (LIN, %), and amplitude of the lateral head displacement (ALH, μm). The percentage of total motility (VAP > 10 μm/s) and progressive motility (VAP > 10 μm/s and STR > 70%) were evaluated based on the default settings of the CASA software.

#### 2.4.2. Sperm Mitochondrial Integrity

Aldehyde fixable MitoTracker Red CMXros (#M7512, ThermoFisher Scientific, Waltham, MA, USA) was used for sperm mitochondria integrity analysis simultaneously with staining by DAPI (4′,6-diamidino-2-phenylindole; Vector Laboratories, Burlingame, CA, USA) to distinguish the sperm from debris or other particles in the extender. After dilution of the sperm to a concentration of 1–10 × 10^6^/mL, 5 µL of MitoTracker Red CMXros working solution (prepared according to the manufacturer’s recommendations) was added and incubated in the dark for 15 min. The samples were then washed and fixed with 3% methanol-free paraformaldehyde. Before flow cytometry analysis, the DAPI dye was added to reach a final concentration 0.5 µg/mL.

#### 2.4.3. Simultaneous Analysis of Sperm Viability and Acrosome Integrity

For analysis of the sperm viability, a fixable Zombie Dye kit (#423107, Biolegend, San Diego, CA, USA) was used according to the manufacturer’s protocol. For acrosome damage detection, lectin Peanut Agglutinin (PNA) conjugated with AlexaFluor 568 (#L32458, ThermoFisher Scientific, Waltham, MA, USA) was used. After dilution to a concentration of 1–10 × 10^6^ sperm/mL in DPBS, the suspension was washed two times at 300× *g* for 10 min/RT, and the pellet was resuspended in 250 µL DPBS. Then, 2.5 μL of a Zombie Dye stock solution (prepared according to the manufacturer’s protocol) and 5 µL of a PNA stock solution (1 mg/mL) were added and incubated for 20 min. The samples were then washed and fixed for 10 min with 3% paraformaldehyde (methanol-free) and analyzed by flow cytometry.

#### 2.4.4. Analysis of Sperm DNA Integrity

The sperm DNA was analyzed by a TUNEL assay using an Apo-Direct Apoptosis Detection Kit (#88-6611-88, ThermoFisher Scientific, Waltham, MA, USA). After thawing and dilution of the sperm to a concentration of 1–10 × 10^6^/mL, 1 mL of chilled Intracellular Staining Perm Wash Buffer (Biolegend, San Diego, CA, USA; prepared according to the manufacturer’s protocol) was added. The sample was gently resuspended with a pipet tip and incubated for 45 min at 4 °C. After washing two times in PBS, the sperm pellet was resuspended in 70% ethanol and incubated for 30 min at 4 °C. Then, the samples were washed two times using the wash buffer included in the kit, and a cocktail of reagents was prepared freshly according to the manufacturer’s recommendations (10 µL reaction buffer, 0.75 µL TdT enzyme, 8 µL FITC dUTP, and 34 µL Milli-Q water) and added. Incubation was carried out for 60 min at 37 °C with shaking. Afterward, the samples were washed three times, and 500 µL of RNAse-free propidium iodide (PI) was added (included in the kit). After 30 min, the incubation samples were analyzed by flow cytometry.

### 2.5. Flow Cytometry Analysis

Flow cytometry analyses of the sperm samples were performed using a BD LSRFortessa TM SORP instrument (Becton Dickinson, San Jose, CA, USA). The following lasers and filter parameters were selected, according to which a fluorescent probe was used: FITC blue laser = 488 nm (100 mW) excitation and 530/30 emission filter; propidium iodide and mitotracker red CMXros = 561 nm (50 mW) laser line excitation and 586/15 nm emission filter; and Zombie viability dye and DAPI = 405 nm (50 mW) laser line excitation and 450/50 nm emission filter.

The voltages were set for the optimum resolution, and minimally, 20,000 gated events (based on FSC and SSC) were recorded for each sample. As a standard procedure before each specific sperm parameter evaluation, positive and negative control samples were prepared to ensure the correct setting of the flow cytometer.

### 2.6. Statistical Analysis

The individual sperm kinematic parameters were analyzed by k-mean cluster analysis to classify the motile spermatozoa into subpopulations. The Euclidean distances algorithm processed the variables STR, VAP, VCL, VSL, and ALH, with 20 iterations used to define three clusters (subpopulations) of sperm. According to the computed means of the selected variables, individual spermatozoa were assigned afterward to one of three specific sperm subpopulations: fast, medium, and slow. To determine the differences in the distribution of these subpopulations, the χ^2^ test was used. The remaining qualitative parameters were analyzed by one-way ANOVA, and any significant differences with the level of significance (*p* < 0.05) were determined using a Tukey post hoc test.

## 3. Results

### 3.1. In Silico Study of Sperm Surface Protein Interaction with Dextran and Pentaisomaltose

**Figure 1:**
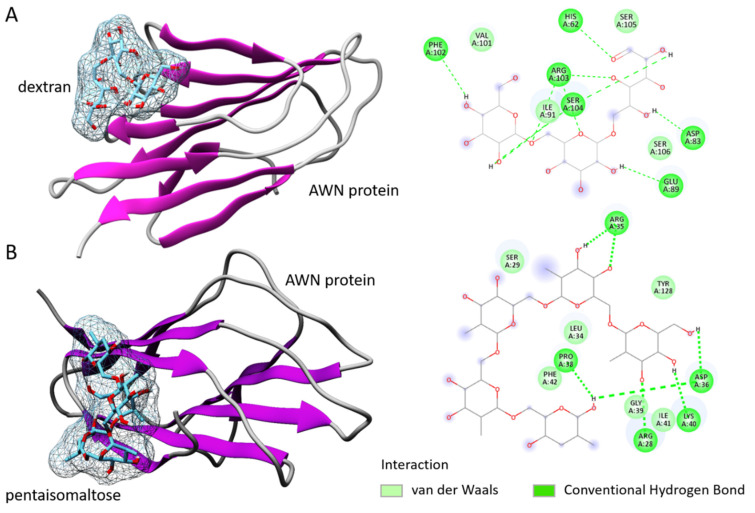
Protein–ligand interaction study. (**A**) Dextran in interacting mode with AWN spermadhesin with specified and interacting amino acids. (**B**) Pentaisomaltose in interacting mode with AWN spermadhesin with specified and interacting amino acids.

The interaction of each polysaccharide compound with the AWN protein is presented in Figure 1. Dextran-AWN showed a moderate binding energy (−5.0 kcal/mol). The results showed eight hydrogen bonds with six main protein residues: His62, Asp83, Glu89, Phe102, Arg103, and Arg104. Dextran also showed one Van der Waals interaction with Ile91, the residue of the AWN spermadhesin (Figure 1A). Analysis of the pentaisomaltose-AWN interaction showed a higher binding energy (−5.9 kcal/mol) but still within a moderate range. The results revealed hydrogen bonds with five protein residues—Arg35, Pro38, Asp36, Arg28, and Lys40—and Van der Waals interactions with Ser29, Tyr128, Leu34, Phe42, Gly39, and Ile41. For proper complexity for the results, the protein–ligand stability and dynamics were studied by MD simulation.

### 3.2. MD Simulation Analysis

#### 3.2.1. RMSD Analysis

**Figure 2:**
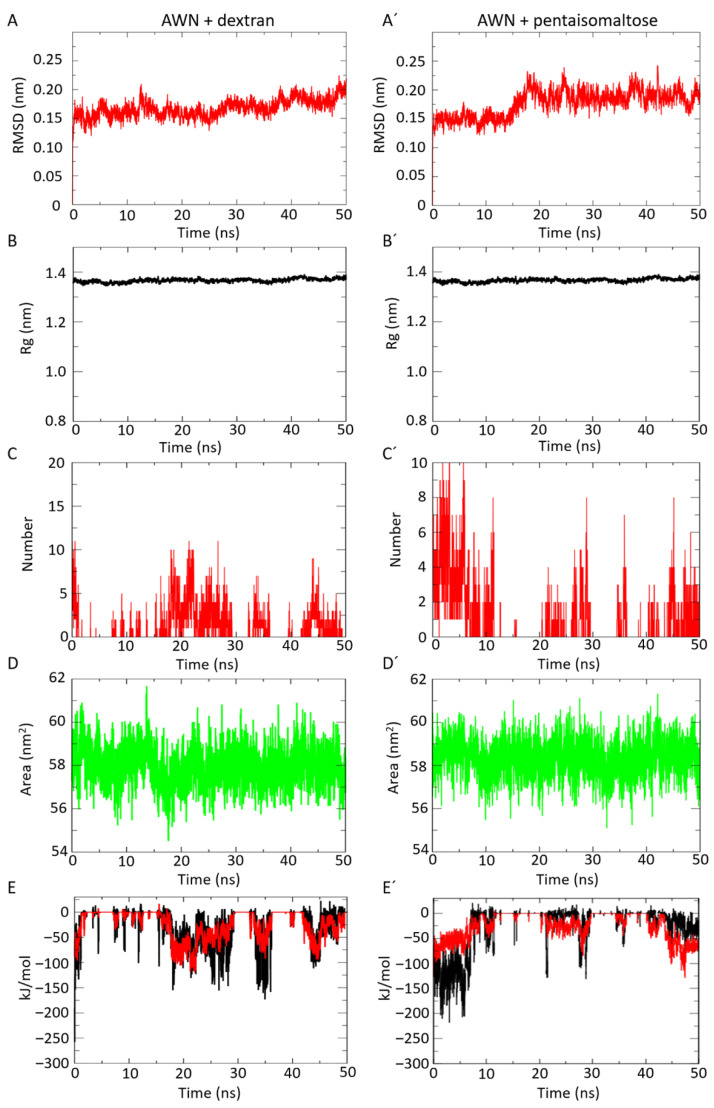
Molecular dynamics simulation analysis of AWN spermadhesin with dextran (**A**–**E**) or pentaisomaltose (**A′**–**E′**). (**A**,**A′**) Protein–polysaccharide complex RMSD plot showing very stable interaction. (**B**,**B′**) Radius of gyration (Rg) plot for protein-polysaccharide complex showing the higher structural compactness of AWN spermadhesin in the presence of dextran or pentaisomaltose. (**C**,**C′**) Plot for H bonds for protein–polysaccharide interacting complex showing six stable hydrogen bonds. (**D**,**D′**) Solvent accessible surface area (SASA) plot for protein–polysaccharide complex. (**E**,**E′**) Short-range electrostatic (Coul-SR) (black line) and Van der Waals or hydrophobic (LJ-SR) interaction energies (red line).

The in silico docking study clearly indicated moderate binding affinity for dextran and pentaisomaltose with AWN spermadhesin. To determine the AWN-docked complex conformation stability with both polysaccharides, the backbone RMSD was computed. The results show that the RMSD trajectory of AWN spermadhesin-dextran was equilibrated over 0–5 ns and remained steady with an RMSD value ∼1.8 ± 0.2 Å at the end of the simulation at 50 ns (Figure 2A), which indicates very stable structural complexity for the AWN spermadhesin–dextran complex. The RMSD trajectory of AWN spermadhesin with pentaisomaltose was equilibrated over 0–10 ns and remained steady with an RMSD value ∼1.8 ± 0.1 Å at the end of the simulation at 50 ns (Figure 2A′), indicating high structural stability. The RMSD values indicate a stable structural conformation of the AWN spermadhesin with dextran or pentaisomaltose without any fluctuations. The RMSD value supports that there can be moderate affinity of the dextran or pentaisomaltose with AWN spermadhesin, but in the presence of dextran, the structural complexity of AWN spermadhesin was stable.

#### 3.2.2. Radius of Gyration (Rg) Analysis

The conformation stability of the AWN spermadhesin with selected polysaccharides was evaluated by the Rg. In this case, the Rg parameter was used to describe the structural compactness of the proteins. To examine the structural compactness and integrity of the AWN–dextran-bound complexes, the radius of gyration (Rg) was calculated for each system [35,36]. The structural compactness of both the AWN–dextran and AWN–pentaisomaltose complexes calculated by Rg analyses suggested stable molecular interaction with all compounds, which were stabilized in 14 Å ± 0.1 Å (Figure 2B,B′).

#### 3.2.3. Hydrogen Bond Analysis

The time evolution plot of the hydrogen bond (H-bond) occupancy between the targeted AWN spermadhesin and dextran or pentaisomaltose was analyzed. H bonds are also designated as the “master key of molecular recognition” due to their crucial role in ligand binding and enzyme catalysis. In the case of AWN spermadhesin–dextran interactions, initially, 10 H bonds were detected. Finally, at 50 ns, 6 H bonds were detected (Figure 2C), and this closely supports our docking interaction data, where we found 7 H bond interactions. Similarly, for AWN–pentaisomaltose, after 50 ns, 6 H bonds (Figure 2C′) were noticed, which was similar to and supported the docking interaction data (7 H bonds).

#### 3.2.4. Solvent Accessible Surface Area (SASA) Analysis

The protein conformational dynamics also depends on hydrophobic interactions. The appropriate functioning of protein–ligand complexes depends on how well the protein maintains its fold during these interactions. Our SASA analysis (Figure 2D) shows that the complex structure AWN spermadhesin occupied with dextran or pentaisomaltose had an average SASA value of 58 nm^2^ ± 1 nm^2^ and 59 nm^2^ ± 1 nm^2^, respectively. In our study, we found that the AWN protein had very high hydrophobicity and a less accessible surface for each polysaccharide binding, which was also reflected in our previous wet-lab experiments and docking study. Thus, the SASA investigation for the protein–ligand complex suggested no significant changes in the conformational dynamics of AWN–dextran- or pentaisomaltose-forming complexes.

#### 3.2.5. Interaction Energy (Coul-SR and LJ-SR) Analysis

The short-range electrostatic (Coul-SR) and Van der Waals or hydrophobic (LJ-SR) interaction energies between spermadhesin AWN and each polysaccharide explained the moderate electrostatic and low hydrophobic interactions. For AWN–dextran, the average values of Coul-SR were −24.15 ± 1.2 kJ/mol, and LJ-SR values of −25.36 ± 2.9 kJ/mol were observed (Figure 2E). Analysis of AWN–pentaisomaltose revealed average Coul-SR and LJ-SR values of −24.92 ± 1 kJ/mol and −25.80 ± 1.9 kJ/mol, respectively (Figure 2E). These interactions suggested that the role of short-range electrostatic interaction and hydrophobic interaction played the same role in AWN–dextran and AWN–pentaisomaltose complex formation.

### 3.3. In Vitro Testing of the Effect of Dextran and Pentaisomaltose on Sperm Qualitative Parameters

Sperm samples for cryopreservation were prepared in the following experimental groups: G3 (control with 3% of glycerol), G2D1 (10) (2% glycerol, 1% dextran 10 kDa), G2D1 (20) (2% glycerol, 1% dextran 20 kDa), G1P5 (1% glycerol, 5% pentaisomaltose/PentaHibe^®^), and G0P10 (0% glycerol, 10% pentaisomaltose/PentaHibe^®^).

**Figure 3:**
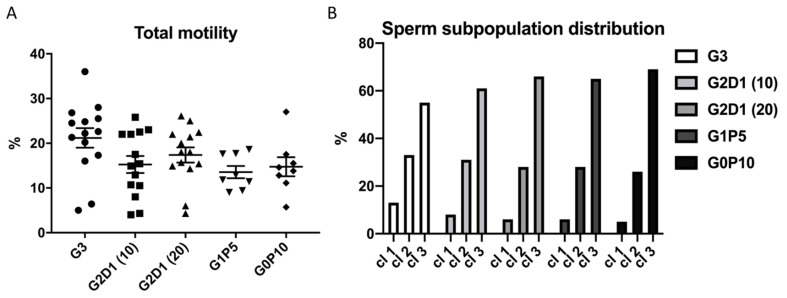
Boar sperm motility analysis after cryopreservation in modified extender. (**A**) Total sperm motility and (**B**) distribution of sperm subpopulations after cryopreservation in extender modified by dextran and pentaisomaltose as G3 (control with 3% of glycerol), G2D1 (10) (2% glycerol, 1% dextran 10 kDa), G2D1 (20) (2% glycerol, 1% dextran 20 kDa), G1P5 (1% glycerol, 5% pentaisomaltose), and G0P10 (0% glycerol, 10% pentaisomaltose) for cl1 (fast sperm subpopulation), cl2 (medium sperm subpopulation), and cl3 (slow sperm subpopulation). Semen from four boars were collected, and each individual was analyzed in three replicates. Statistical significance (*p* < 0.05) was not found.

After thawing the sperm, the first analyzed parameter was their motility (CASA). The sperm motility, evaluated as a percentage of the total motile sperm (Figure 3A), was affected neither by dextran nor by pentaisomaltose (*p* > 0.05) (Figure 3A). Based on cluster analysis of the individual sperm kinematic parameters, the following sperm subpopulations were defined: fast (cl1), medium (cl2), and slow (cl3). Deeper insight into the sperm motility by k-mean cluster analysis did not show significant differences between the experimental groups of sperm relative to the proportion of fast, medium, and slow clusters (Figure 3B).

**Figure 4:**
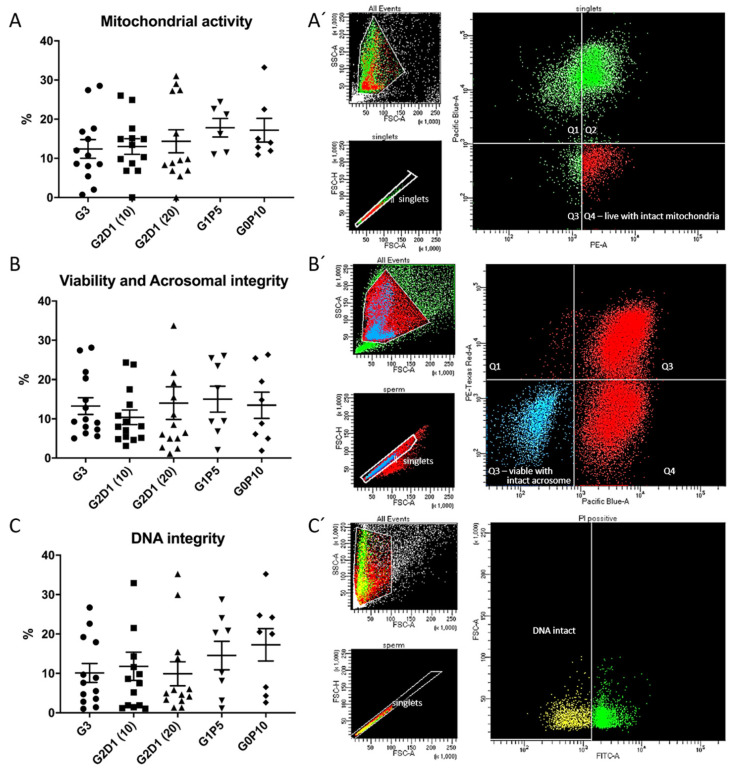
Results of flow cytometry analysis of sperm qualitative parameters after cryopreservation in modified semen extender by dextran and pentaisomaltose. (**A**) Mitochondrial activity evaluation. (**A′**) Representative dot plot of sperm mitochondrial activity analysis by simultaneous staining with Zombie Viability Dye (UV laser Pacific Blue) and Mitotracker Red CMXros (yellow-green laser PE), where the population of interest is in Q4 (red color). (**B**) Viability and acrosomal integrity evaluation. (**B′**) Representative dot plot of sperm viability (Zombie Viability Dye; UV laser, Pacific Blue) and acrosomal integrity (lectin PNA-AlexaFluor 568; yellow-green laser, Texas Red) analysis. Population of viable sperm with intact acrosome is in Q3 (blue color). (**C**) DNA integrity evaluation. (**C′**) Representative dot plot of sperm DNA integrity analysis performed by TUNEL assay (blue laser, FITC). Population of sperm gated by positive propidium iodide signal with intact DNA is highlighted in yellow for G3 (control with 3% of glycerol), G2D1 (10) (2% glycerol, 1% dextran 10 kDa), G2D1 (20) (2% glycerol, 1% dextran 20 kDa), G1P5 (1% glycerol, 5% pentaisomaltose), and G0P10 (0% glycerol, 10% pentaisomaltose). Semen from four boars was collected, and each individual was analyzed in three replicates. Statistical significance (*p* < 0.05) was not found.

In all experimental groups, we analyzed other sperm parameters such as mitochondrial activity, viability together with acrosomal integrity, and DNA integrity by flow cytometry. In the case of the first analyzed parameter, significant differences (*p* > 0.05) were not found (Figure 4A). The viability and acrosomal integrity of the sperm after thawing were not significantly affected (*p* > 0.05), with higher variability of results in individual groups, including the control (Figure 4B). The results for sperm DNA integrity evaluated by a TUNEL assay revealed no significant differences, but there was an interesting effect from pentaisomaltose in the group with complete substitution of glycerol (G0P10), which is visible in Figure 4C.

## 4. Discussion

Cryopreservation of sperm is one of the most efficient ways to ensure sustainable production of long-term insemination doses for commercial purposes and to maintain the genetic diversity of autochthonous livestock breeds and wildlife species. We are the first to publish a possible way to modify the boar semen extender using dextran or its side product pentaisomaltose, which are already used clinically in the cryopreservation of other cell types [29]. Our in vitro testing of the efficiency of the selected compounds was coupled with an in silico study with computational modeling. The last approach is unique in reproductive biology and represents a new method for the prediction of interactions between different biological compounds with targeted proteins on the sperm’s surface.

Boar spermatozoa are one of the most sensitive sperm types to cryopreservation. The resistance of spermatozoa to cryopreservation is highly dependent on their biophysical properties such as the shape, size, and plasma membrane composition, which is mainly linked to a lower cholesterol content and high content of polyunsaturated fatty acids [37,38,39].

Because of species-specific gamete sensitivity, it is almost impossible to develop a standard cryopreservation protocol that will cover most species [40]. It is crucial to continue with testing and studying other compounds that have potential benefits for cryopreservation protocols. Generally, it is agreed that in relation to the speed of freezing, the most important factor is intracellular ice formation. However, there is no clear evidence of ice’s presence in the absence of glycerol in cryopreservation media. Morris [41] showed that in the case of human sperm, there is no evidence of intracellular ice crystal formation even with a rapid cooling rate. In the mouse model, no direct evidence of intracellular ice in the sperm head was found [42]. Indeed, improving the extracellular environment is therefore one of the most important approaches for reducing the physiologically harsh conditions for sperm during cryopreservation, thus leading to better recovery rates after thawing.

Dextran is an α-D-1,6-glucose-linked polymer, with sidechains 1–3 linked to the backbone units. Regarding spermatozoa, it was hypothesized [43] that dextran works as a cryoprotectant during ice crystal formation. Dextran has many –OH groups and thus may form a layer on the sperm surface by forming H bonds with the phosphate head groups of the membrane phospholipids [44]. Our in silico study revealed that the dextran and its side product pentaisomaltose had moderate binding affinities toward AWN proteins from the spermadhesin family, which are present on the surface of boar spermatozoa [45]. This was further supported by in silico molecular docking, protein–ligand interaction, and an MD simulation study. In silico modeling data of dextran and pentaisomaltose with AWN spermadhesin showed only weak hydrogen bonds and Van der Waals interaction without any covalent binding. The results of the docking analysis showed a similar binding energy score, indicating moderate protein–ligand affinity. However, the energy for the AWN–pentaisomaltose complex was calculated to be higher (−5.9 kcal/mol) than for the complex with dextran. The RMSD value without any fluctuations supports that the pentaisomaltose had very stable interactions with AWN spermadhesin.

The interaction study together with the MD simulation suggests the high stability of both polysaccharides (dextran and pentaisomaltose) interacting via several amino acids of the AWN protein and making a protective layer on the sperm’s surface. Additionally, docking analysis showed a hydrophobic pocket in the molecule of pentaisomaltose for the AWN protein, and compared with dextran, different AWN spermadhesin amino acid residues interacted with pentaisomaltose. Importantly, our previous experiments proved the binding ability of AWN spermadhesin (specifically isoform AWN-1) with some polysaccharides, including dextran sulphate [23,24]. There is a possibility that due to the reaction of AWN protein, dextran could hypothetically mask sites on the sperm’s surface, which is important for the interaction with oviductal proteins after insemination dose deposition to the female reproductive tract. Nevertheless, AWN protein alone has not been described as a binding partner for oviductal epithelial molecules [46]. In the future, it will be necessary to focus experiments on the accessibility of important epitopes on the sperm surface after their thawing, together with the evaluation of the capacitation process.

The high number of –OH groups in dextran interfering with H bond formation inhibits large ice crystal formation during semen cryopreservation [47]. Moreover, it was reported that aqueous solutions with polymers reduce the crystallization temperatures, and finally they can undergo supercooling more easily [48]. There are currently only two recent studies focusing on the elimination of glycerol by dextran from cryopreservation extenders [47,49]. The results of the study performed by Gloria et al. [47] showed the possibility of complete substitution of glycerol with dextran for turkey sperm. They found that certain concentrations of dextran in the medium had a positive effect on the functional parameters and fertilizing ability in vitro of sperm after thawing. The presence of glycerol in cryoprotective medium for the storage of avian sperm causes almost 100% inhibition of the fertilization capacity [50]. This study inspired us to test this very promising alternative for boar sperm cryopreservation media improvement. A very recent study [49] showed the possibility of using dextran in rabbit sperm cryopreservation. However, there are discrepancies in the motility results and other qualitative parameters, such as the outcome of in vitro fertilization.

Although the results of media modification did not significantly affect the motility of the sperm after thawing, there was a trend in the increase of maintained mitochondrial activity throughout all experimental groups, where the use of pentaisomaltose (groups G1P5 and G0P10) showed more consistent results. It can be hypothesized that there could be interactions by dextran and pentaisomaltose with proteins linked to energy metabolism [51]. In the case of AWN–polysaccharide interaction, this can be explained by the fact that AWN is found in a majority on the sperm’s surface [45]. The interaction of these polysaccharides with plasma membrane proteins may also lead to better mitochondria recovery after thawing. The beneficial effect of dextran and pentaisomaltose can also be seen in a certain level of DNA integrity improvement. Interestingly, this effect was most prevalent when pentaisomaltose was used for the entire substitution of glycerol. Glycerol is a toxic agent that leads to harmful changes in spermatozoa, including protein changes and actin alterations [16]. The effect on viable sperm with intact acrosomes was inconsistent. In the experimental group with an absence of glycerol (G0P10), the values were the lowest, suggesting the essential role of glycerol in protectivity of such fragile sperm head organelles as acrosomes. Additionally, pentaisomaltose has been used in experiments for improving the cryopreservation protocol of human hematopoietic stem cells (HPCs) [30]. These HPCs were further used for successful transplantation, and thus no toxicity was confirmed. Moreover, it showed a higher rate of recovery and functionality of HPCs in comparison with DMSO, which also showed a certain level of toxicity in the case of boar spermatozoa [52]. In this paper, we reported for the first time the use of pentaisomaltose in sperm cryopreservation. Our experiments showed it has promising potential as a substitute for glycerol in the extender for semen cryopreservation. This finding is supported by affinity modeling of pentaisomaltose binding to AWN spermadhesin located on the sperm’s surface. Moreover, it might be proposed that the positive effect of the pentaisomaltose is in the improved maintenance of osmotic conditions [31].

Controversial results are continuously reported regarding post-thaw sperm survival and quality, as well as the impact of sperm cryopreservation on reproductive outcomes following artificial insemination [53]. Artificial insemination with cryopreserved semen has a positive impact on porcine production and product quality. Multiple seminal doses of boar sperm with high genetic merit can be obtained from one ejaculate, and storage and transport in liquid nitrogen allow the insemination of thousands of sows worldwide [38]. Another essential use is for the creation of genetic resource banks which preserve the genetic material of endangered, valuable individuals or genetically relevant breeds [54]. The need to preserve genetic variability in domestic livestock has been recognized for many years [55]. These breeds present characteristics not found in the more productive and selected commercial breeds, such as rusticity, adaptation to particular environments, good maternal qualities, longevity, and disease resistance, which are of great interest in a world facing environmental challenges [56,57]. Additionally, they are also culturally and historically valuable, meriting preservation for further generations [58]. The preservation of genetic pools is crucial for animal conservation because it prevents the loss of genetic diversity, an essential factor for protecting populations during unforeseen situations [54]. Due to this reason, our research to find the most suitable cryoprotectants can be applied to Prestice black-pied pigs, which are part of the Czech governmental program for national genetic resource preservation.

## 5. Conclusions

The main objective of our study was to test the potential for a reduction in the glycerol concentration in the boar semen extender, resulting in a possible improvement in the sperm survival rate after cryopreservation by selected polysaccharides (dextran and pentaisomaltose). In contrast to dextran, the in vitro experiments demonstrated a promising effect of pentaisomaltose as a complete substitute for glycerol in the freezing extender for boar semen. An in silico study was performed to provide a deeper insight into the protective mechanism of the selected polysaccharides. This study proved the stable interaction of dextran and pentaisomaltose with the sperm surface protein AWN spermadhesin. A higher binding affinity was shown to occur with the pentaisomaltose–protein complex. Finally, this unique experimental approach of coupling in silico and in vitro testing in cryopreservation represents an excellent tool for the prediction of novel cryoprotectants and an innovative approach toward the selection of new substances for further applicational studies.

## Data Availability

The data presented in this study are available on request from the corresponding author.
